# Improved Gait Parameters After Orthotic Treatment in Children with Infantile Tibia Vara

**DOI:** 10.1038/s41598-020-59599-8

**Published:** 2020-02-21

**Authors:** Serap Alsancak, Senem Guner, Hakan Kınık

**Affiliations:** 1 Department of Prosthetics and Orthotics, Faculty of Health Sciences, Ankara University, Ankara, Turkey; 2 Department of Orthopedics and Traumatology, Faculty of Medicine, Ankara University, Ankara, Turkey

**Keywords:** Paediatric research, Rehabilitation

## Abstract

The aim of this study was to investigate the modification of gait kinematics before and after orthotic treatment in patients with ITV. Vicon instrumented gait analysis was performed on three patients with ITV, pre and post treatment. Orthoses were applied a total of eighteen participants with ITV who were 25–38 months. 34 extremities were treated and radiographic evidence evaluated before and after orthotic treatment. Treatment duration for orthotic treatment ranged between 11 and 41 (25.9 ± 10.0) months. Only three patients were evaluated in gait analysis due to application difficulties. Three patients kinematic and kinetic instrumented gait analysis were found flatfoot, varus and internal rotation of the foot, hip flexion and external rotation. Study were reported an improvement in gait kinematics after orthotic treatment, in patients with ITV.

## Introduction

Studies on infantile tibia vara (ITV) have been performed since the 1960s^1–5^, with surgical correction, orthotic applications, and spontaneous natural healing; most studies performed radiographic evaluations, which demonstrated metaphyseal-diaphyseal proximal tibial angles with progression of distal femoral deformities^2,6,7^. In 1952, Langeskiöld classified ITV into six stages, based on the degree of metaphyseal-epiphyseal changes seen on radiographs; variations occurred with advancing age. Two forms of the deformity are recognized based on age at onset of the condition such as infantile form, if younger than 3 years, and adolescent form, if 10 years or older^1,2^. Early ambulation and body weight were determined to contribute to the development of ITV, which is not seen in non-ambulatory patients. Ligamentous laxity and lateral thrust of the knee during the stance phase of gait have also been cited as determining factors^1,8^, and abnormal increases in knee internal rotation and hip external rotation moments have been observed^9^. Blount disease affects the proximal tibia and also involves the distal and proximal femur and ankle to varying degrees. In contrast, femoral anteversion is often increased. The sole of the foot usually lies flat on the ground during the stance phase, despite varus deformity due to concomitant talocrural joint deformity combined with medial rotation of the leg bones^10^.

The nonsurgical treatment of ITV has been controversial. However, recent studies have indicated success when a knee ankle foot orthosis (KAFO) was used nearly full-time to improve biomechanics in patients aged <4 years^11–13^. The efficacy of orthoses with corrective forces and/or distraction systems is related to their ability in relieving weight-bearing stresses on the medial physeal region of the proximal tibia.

Alsancak *et al*. reported that a single upright KAFO with a drop lock knee joint was effective for the treatment of ITV in 20 children aged up to 38 months^11^. The treatment duration in that study extended for nearly 6 months. Whiteside *et al*. reported that in 2 children aged approximately 46 months, double, solid, upright KAFOs with free knee motion significantly improved ITV after 17 months of treatment^12^. Single or double upright KAFOs that apply four or five corrective forces or a distraction system, or variations of these devices, have been described in the orthotic management of ITV. This study aimed to determine the pre- and post-orthotic treatment effects in early-stage ITV using three dimensional gait analysis.

## Methods

### Participant characteristics and ethical considerations

This study included 18 participants with ITV between the ages of 25 and 38 months (mean ± standard deviation [SD], 31.6 ± 3.5 months) and percentile(BMI) 50 and <97 (83.6 ± 15.7) who were referred by an orthopaedic surgeon to the University Prosthetics and Orthotics Laboratory to receive orthotic treatment within a year. Approval was obtained from the ethical board committee of Ankara University and all research was performed in accordance with relevant guidelines/regulations. Informed consents were obtained from parent before any study procedures were initiated.

**Figure 1:**
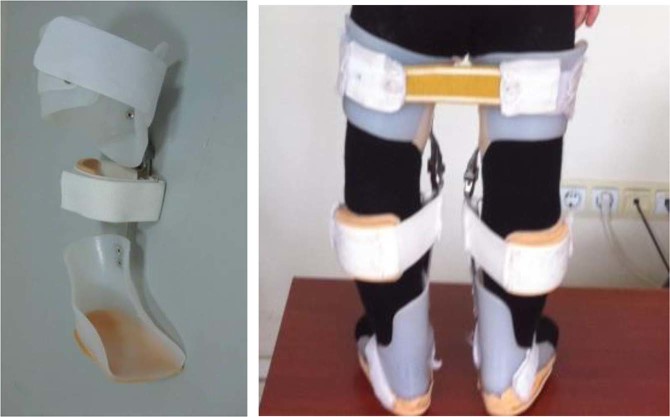
KAFO and application patient with bilateral ITV.

Radiographic diagnosis of each child participant prior to orthotic use was performed according to the Langenskiöld criteria, which is used for ITV. According to this classification, most of the participants were classified as Langenskiöld Stage I and II. Patients were fitted bilaterally with single medial, upright metal KAFOs with drop lock knee joints. The orthoses applied five constant corrective forces to the femur and proximal tibia, and the lateral corrective band that was applied across the tibia produced a rigid column (Fig. 1)^11^.

Three patients with ITV (Langenskiöld Stage II) gait evaluations were performed pre- and post-treatment for both legs (mean ± standard deviation [SD], age; 33 ± 2.6 months). Gait analysis was conducted in a gait laboratory using a Vicon motion analysis system (Vicon Nexus, Oxford Metrics, Oxford, UK) with six infrared cameras at 240 Hz. Each camera and force plate was calibrated before data collection. Data were collected after several practice trials. The average of five trials for each walking condition was calculated. The patient walked a distance of 10 m at a self-selected speed in the gait laboratory before and after treatment.

### Orthotic treatment procedure

**Figure 2:**
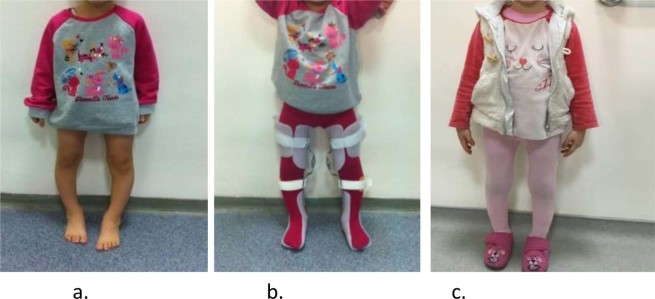
(**a**) Before treatment with patient ITV. (**b**) KAFO with patient ITV. (**c**) After orthotic treatment with patient ITV.

**Figure 3:**
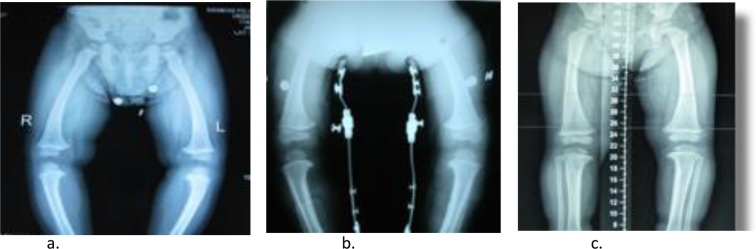
Anteroposterior radiographs of lower extremity, (**a**). Before treatment, (**b**). Before treatment with KAFO, (**c**) After treatment.

**Figure 4:**
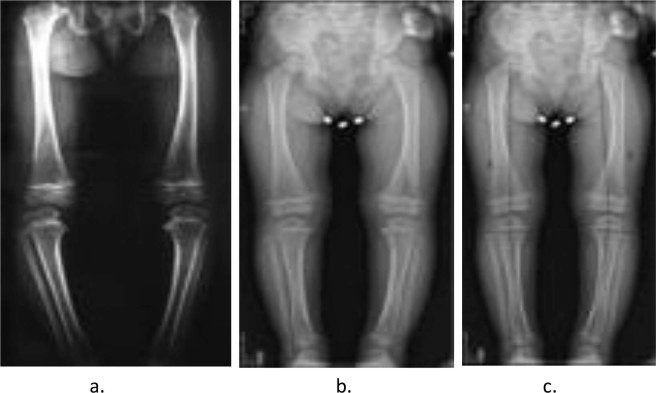
Anteroposterior radiographs of lower extremity, (**a**). Before treatment, (**b**,**c**). After treatment.

In the 18 children with ITV (16 bilateral and 2 unilateral), KAFOs were applied to 34 extremities. Twisters were added to the orthosis of nine children with tightness in the hip rotators. Treatment duration for orthotic treatment ranged between 11 and 41 months (mean ± SD; 25.9 ± 10.0 months) (Fig. 2). Evaluated pretreatment and posttreatment anteroposterior radiographs of lower extremity showed Figs. 3 and 4.

### Factors that shorten the treatment process

It is known that full-time use of orthoses is more effective than night-time use, polypropylene is more effective than polyethylene and knee locking is more effective than free use. Additionally, if children with tibial torsion have rotator tightness, a twister is effective. Moreover, careful follow-up is important in the first 3 months of orthotic treatment, as rapid tibial growth is reflected in the orthosis in the first 3 months of follow-up and necessary modifications are very effective in the treatment process, as well as the planned exercise program with the orthosis.

### Disadvantages in the treatment process

Disadvantages include restricting activity of the child during the day, prolongation of the treatment period due to the family’s choice of night-time use only and orthosis is faster than the horizontal corrective effect of the frontal plane. In addition, polyethylene is preferred for the medial upright bar of the KAFO due to breakage that tears the plastic around the rivet.

### Statistical analysis

Statistical analyses were performed using SPSS (version 16; SPSS, Inc., Chicago, IL, USA) and using descriptive analysis test was performed. As the number of cases with three dimensional (3D) was a small group, 3D gait analysis were not analysed statistically.

## Results

**Figure 5:**
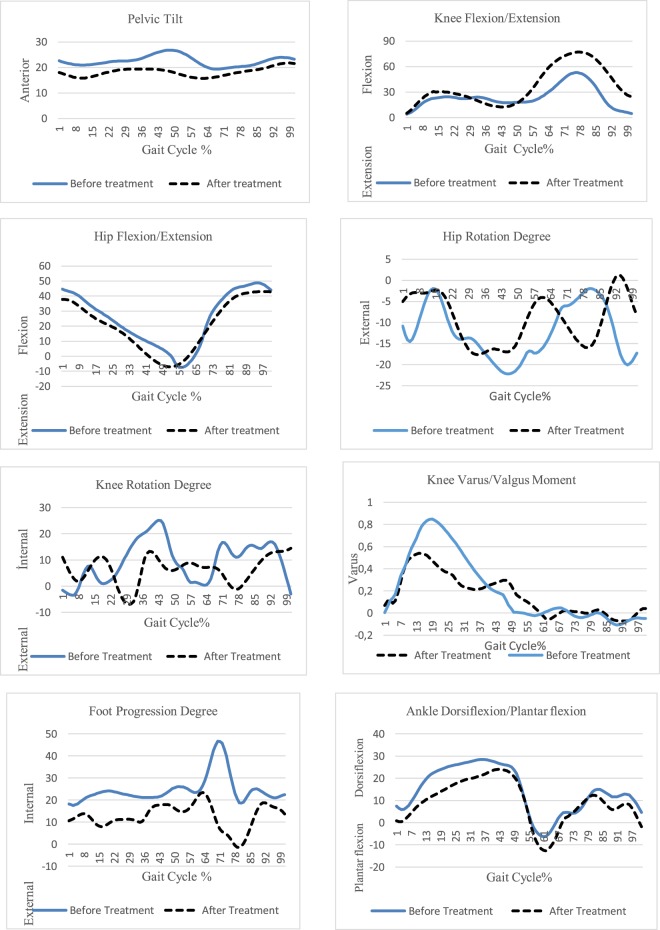
Kinetic and kinematic gait analysis results in ITV patients.

3D analysis test results are shown in Table 1. Before treatment results of gait analysis graphics showed flatfoot (total foot base contact) and foot internal progression degree in loading response phase, increased knee varus moment, anterior pelvic tilt and increased hip external rotation in stance phase with three ITV patients during gait cycle (Fig. 5). According to the results of the gait analysis graphics, flatfoot continues after treatment.

**Table 1:** Comparison of before and after orthotic treatment of gait analysis results.

Parameters	Before Orthotic Treatmet	After Orthotic Treatment
**Pelvic Tilt**		
Max. Anterior Pelvic Tilt (°)	26.6	19.3
**Hip**		
Max. Hip Extension (°)	−7	−6.3
Max. Hip Flexion (°)	43.5	37.6
Max Hip External Rotation (°)	−22.1	−16.9
**Knee**		
Max. Knee Extension (°)	12.5	12.7
Max. Knee Flexion (°)	52.9	75.3
Max Knee İnternal Rotation(°)	25.1	12.5
Knee Varus Moment (N/kg)	0.83	0.53
**Ankle**		
Max. Ankle Dorsi Flexion (°)	28.3	24.6
Max. Ankle Plantar Flexion(°)	−6.2	−12.6
Foot Progression Degree	26	17.4

Before treatment, the foot progression degree was internally rotated that was decreased after treatment. Furthermore, before treatment, the pelvis was in the down position and had protraction and the trunk was moderately backwards during the terminal stance phase of the gait cycle; the pelvis and trunk kinematics were considered improved after treatment. According to result of gait analysis graphics were decreased foot progression degree, knee varus moment and pelvic anterior tilt degree after orthotic treatment. The results of the kinematic and kinetic analysis of the ankle, hip and knee joint walking parameters are shown in Graphic 1. Before treatment, the lower extremity kinematic assessments indicated knee internal rotation and degrees of hip external rotation were increased during late stance phases. After treatment, hip and knee rotation decreased in gait analysis graphics.

The difficulties of evaluating children by gait analysis is quite high. In our study, results of gait analysis was in terms of giving general information about walking assessment of ITV patients in clinic.

## Discussion

**Figure 6:**
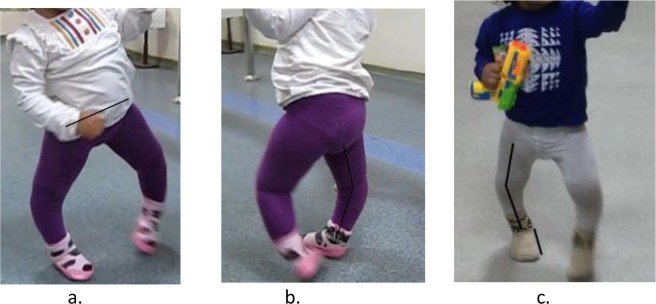
(**a**) Pelvic obliquity at mid-stance. (**b**) Knee hyperextension in stance. (**c**) Foot internal rotation in stance.

According to the visual gait assessment in children with ITV, pre-treatment pathological gait patterns, such as standing flatfoot, varus and internal rotation of the foot, internal rotation of the knee, hip flexion and downward position and protraction of the pelvis, during the stance phase became normalized or decreased post-treatment (Fig. 6). Despite these findings, treatment with orthosis in patients with ITV remains debatable.

By age 2 years, there is more clearly defined knee-flexion wave and heel strike contact, decreased hip external rotation and increased hip adduction in stance^14^. In our study, patients with ITV exhibited poor kinematics prior to orthotic treatment for example, there was pressing of the entire lateral side of the sole of the foot, flatfoot and inadequate heel lift and toe-off phases during the gait cycle. Additionally, knee progression occurred during lateral displacement of the line, and knee flexion and internal rotation occurred while walking. Simultaneously, while the trunk was performing lateral flexion, pelvic anterior tilt, obliquity and pelvic rotational movement increased, and lumbar lordosis increased with trunk extension. As such, significant improvements in the impaired gait pattern were observed due to the correction of deformity after orthotic treatment.

The pathophysiology of ITV is believed to be related to biomechanical overloading of the posteromedial proximal tibia during gait, with the knee in a varus deformity. Studies have demonstrated considerable rates of spontaneous resolution of ITV staged < Langenskiöld III, indicating that patients were successfully treated with orthoses. Most specialist evaluations reported that a mature gait is present in healthy children by age 5.

However, Sutherland *et al*. reported that a mature gait pattern is established in most children by the age of 4 years^14,15^; they evaluated temporal and distance parameters among 309 healthy children, and suggested that a normal walk ratio and stride length is an idiosyncratic feature of gait between the ages of 7–11 years. Many studies have indicated that treatment using orthoses is effective in the early stages of ITV^16–24^. Raney *et al*., found no difference in clinical improvement between the patients with only night-time and continuous use of orthoses^23^. Montenegro *et al*., found that night-time use of KAFO for medial decompression was effective in reducing the metaphyseal-diaphyseal angle in children with ITV aged under 3 years, irrespective of sex and bilateral disease; patients aged over 3 years did not benefit from bracing^25^. Additionally, we found that KAFO was effective in children aged between 2 and 3.5 years. In patients with ITV, the duration of bilateral orthotic treatment exceeds that of unilateral treatment. Blount advised that his bowleg brace should be used at night in patients aged younger than 2 years^26^. Loder and Johnston showed successful outcomes in 12 of 23 extremities in patients with Blount stage I–II deformities; however, the success rate with orthotic treatment was only 50%^27^. They reported that orthoses were only indicated for children aged between 1.5 and 2.5 years. Full-time use of the locked knee joint orthosis at treatment initiation was an important factor in our study. In addition to this, correction was provided in both, horizontal and frontal planes using orthoses.

In order to determine whether patients treated with orthoses will revert to pathological gait patterns, and to assess the long-term efficacy of orthotic treatment, future research should focus on the long-term follow-up of gait analyses in these patients. Studies using temperature sensors will also be effective in evaluating the follow-up and treatment processes in ITV.

## Conclusion

Our results showed improvements in gait kinematics after orthotic treatment. Assessing gait abnormalities in ITV can be challenging and access to instrumented gait analysis is not always feasible.
